# Case Report: Whole-exome sequencing identified two novel COMP variants causing pseudoachondroplasia

**DOI:** 10.3389/fendo.2023.1267946

**Published:** 2023-11-23

**Authors:** Lin Zhou, Jing Chen, Qian Liu, Shuting Yang, Wanqin Xie, Ying Peng

**Affiliations:** ^1^ Department of Medical Genetics, Hunan Provincial Maternal and Child Health Care Hospital, Changsha, Hunan, China; ^2^ Medical Department, Zhejiang Biosan Biochemical Technologies Co. Ltd, Hangzhou, Zhejiang, China; ^3^ National Health Commission Key Laboratory of Birth Defects for Research and Prevention, Hunan Provincial Maternal and Child Health Care Hospital, Changsha, Hunan, China

**Keywords:** pseudoachondroplasia, comp, whole-exome sequencing, case report, novel missense variants

## Abstract

Pseudoachondroplasia (PSACH) is a rare, dominant genetic disorder affecting bone and cartilage development, characterized by short-limb short stature, brachydactyly, loose joints, joint stiffness, and pain. The disorder is caused by mutations in the *COMP* gene, which encodes a protein that plays a role in the formation of collagen fibers. In this study, we present the clinical and genetic characteristics of PSACH in two Chinese families. Whole-exome sequencing (WES) analysis revealed two novel missense variants in the *COMP* gene: *NM_000095.3: c.1319G>T* (p.G440V, maternal) and *NM_000095.3: c.1304A>T* (p.D435V, paternal-mosaic). Strikingly, both the G440V and D435V mutations were located in the same T3 repeat motif and exhibited the potential to form hydrogen bonds with each other. Upon further analysis using Missense3D and PyMOL, we ascertained that these mutations showed the propensity to disrupt the protein structure of COMP, thus hampering its functioning. Our findings expand the existing knowledge of the genetic etiology underlying PSACH. The identification of new variants in the *COMP* gene can broaden the range of mutations linked with the condition. This information can contribute to the diagnosis and genetic counseling of patients with PSACH.

## 1 Introduction

Pseudoachondroplasia (PSACH) is a rare skeletal dysplasia. This disorder is inherited in an autosomal dominant manner, which means that inheriting a copy of the altered gene from a single parent is sufficient to cause the disorder. The condition is recognized by the Online Mendelian Inheritance in Man (OMIM) database with the MIM number 177170. PSACH is typically characterized by short-limb short stature, brachydactyly, loose joints, joint stiffness, and pain (1). People with PSACH typically have normal growth and development during the first 2 years of life, but then their growth rate slows down significantly and falls below the standard growth curve. Other characteristic features of PSACH include a waddling gait, genu varus, limited elbow and hip range of motion, scoliosis, lumbar lordosis, and spinal stenosis. However, it does not affect craniofacial appearance or intelligence. The severity of these symptoms can vary among affected individuals.

PSACH is caused by mutations in the *COMP* gene (*COMP*; 600310) (2). *COMP* is a member of the thrombospondin gene family and encodes cartilage oligomeric matrix protein, a family of secreted matricellular proteins that modulate cell behavior and ECM organization. (3–5). The COMP protein is composed of multiple domains, including an N-terminal region, a central domain composed of two types of repeats: type II epidermal growth-like repeats (T2 repeats) and type III calmodulin-like repeats (T3 repeats), which are responsible for oligomerization of the protein and its interaction with other ECM proteins, and a C-terminal globular domain (CTD) (6, 7). The T2 repeats, T3 repeats, and CTD can bind calcium ions, which is essential for the correct folding of the protein, and enables it to function properly in cartilage and other connective tissues (8, 9). The loss of tertiary structure and misfolding of the protein due to *COMP* mutations cause substantial retention of COMP in the endoplasmic reticulum (ER), resulting in skeletal abnormalities (such as PSACH or multiple epiphyseal dysplasia) (10, 11).

In PSACH, mutations are mostly found in the T3 repeats whereas mutations in the CTD are less common (6, 7). Interestingly, the position of the pathogenic variant may cause different levels of short stature. Research shows that when the variant occurs in the T3 repeats rather than in other domains, patients may suffer more (12). Specific missense variants in the C-type motif of the T3 repeats result in PSACH, whereas missense variants in the N-type motif generally result in MED (multiple epiphyseal dysplasia), a milder skeletal dysplasia (13). It is worth noting that not all missense variants in the C-type or N-type motifs lead to PSACH or MED. Different mutations that occur at the same site can also lead to different severity of the disease. Individuals carrying p. Asp469del appear extremely short (12), whereas p. Asp469dup results in mild MED (13–15).

In this report, we describe two families with PSACH. All the affected patients had a typical clinical phenotype and were subsequently diagnosed by X-ray. Whole-exome sequencing (WES) identified two novel missense variants in the *COMP* gene. Following the ACMG guidelines, the two variants were interpreted as likely pathogenic. Our findings offer valuable insights into the molecular and clinical characteristics of PSACH among Chinese patients, which can facilitate the early detection of the condition and improve treatment outcomes for individual patients.

## 2 Materials and methods

### 2.1 DNA extraction

The DNeasy Blood & Tissue Kit (Qiagen, Valencia, CA, USA) was used for genomic DNA extraction from peripheral blood samples. Fetal DNA was obtained from amniotic fluid.

### 2.2 Whole-exome sequencing

Agilent SureSelect Human All Exon v.6 was used for targeted exome sequencing library preparation; this method allows capturing all exomes of the human genome for sequencing. Then, high-throughput sequencing was performed on the HiSeq X Ten platform (Illumina Inc., San Diego, CA, USA). Low-quality sequencing reads were filtered out, and the reads were aligned to the NCBI human reference genome (hg19/GRCh37) by Burrows–Wheeler Aligner. The GATK pipeline was used for variant calling, and ANNOVAR for annotation of variants.

### 2.3 Sanger validation

The identified variants were confirmed by Sanger sequencing.

Primer pairs (COMP-e12e13F-L: TGTAAAACGACGGCCAGTGGGAGGCTTTCTGATTTC; COMP-e12e13R-L: CAGGAAACAGCTATGACCAAGTCGTCCTGGCACAC) were used for COMP G440V amplification.

Primer pairs (COMP-18897052-F: TTTCTGATTTCCTCTGTCTGATTATG; COMP-18897052-R: GGGCACTGTTAGGCACCG) were used for COMP D435V amplification.

### 2.4 Predicted structure models

VarSite, Missense3D, and PyMOL were employed to evaluate the functional consequences of the G440V and D435V variants on the COMP protein. The structure of the COMP protein complex was obtained from the Protein Data Bank in Europe (PDBe), providing a basis for analysis. PyMOL was utilized to perform a comparative analysis, make necessary modifications, and visualize the molecular structures of both the wild-type and variant forms of the COMP protein. These tools facilitated a comprehensive examination of the structural changes and potential functional impacts caused by the identified variants.

## 3 Results

### 3.1 Case report

**Figure 1:**
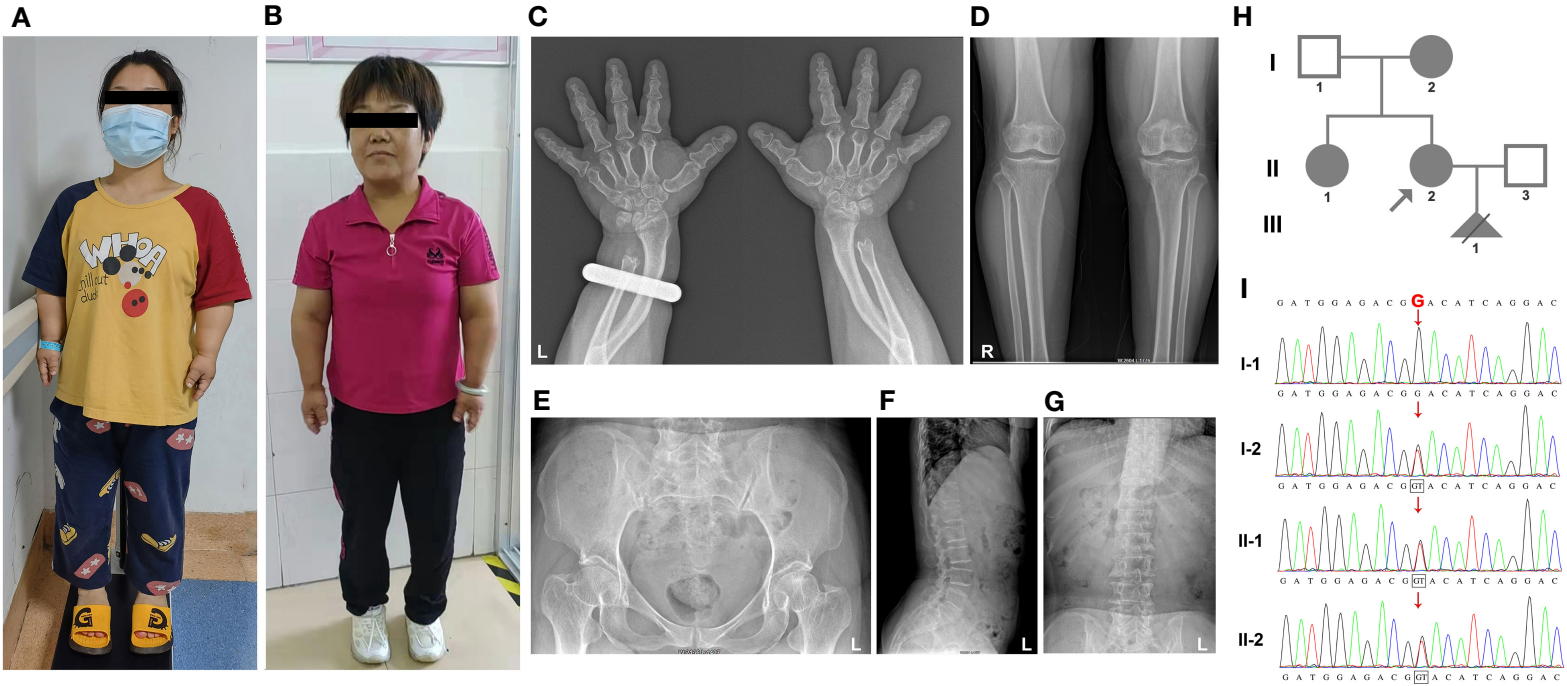
The clinical images, radiological characteristics, pedigree, and co-segregation of the *COMP* mutation in the Family 1. **(A, B)** Clinical photographs show short-limb dwarfism in the proband **(A)** and her mother **(B)**. **(C–G)**, The radiological characteristics of the proband’s mother. Brachydactyly of hands and shorter distal ulna; **(C)** osteoarthritic changes in the knee; **(D)** normal pelvis; **(E)** normal vertebrae and lumbar **(F)**, and no scoliosis **(G)**. **(H)** Pedigree of family 1. **(I)** Consequence of Sanger sequencing.

Family 1: The proband, a 31-year-old pregnant woman with short stature (135 cm), sought genetic and prenatal diagnosis. Her mother and sister exhibited a similar phenotype, characterized by brachydactyly, a waddling gait, genu varum, mild thoracic deformity, and restricted extension at the elbows in the upper limbs (**Figures 1A, B** and **Table 1**). Additionally, the proband’s mother had a degenerative knee joint. Facial features were normal for all affected individuals, and thyroid function tests yielded normal results. Due to the proband undergoing assisted reproductive technology (ART), an X-ray examination was not ordered. However, X-ray images of the proband’s mother revealed brachydactyly of the hands, a short distal ulna, and mild osteoarthritis in the knee joints (**Figures 1C–G**). Based on the clinical presentation, the family was strongly suspected of having PSACH.

**Table 1:** Clinical information of affected family members.

Patient	Family 1			Family 2		
	I-2	II-1	II-2	II-3	III-3	IV-1
Gender	F	F	F	F	F	M
Age	54	26	31	53	28	3
Height (cm)	140	142	135	160	100	79.5
Onset ages (years)	2	2	2	50	2	2
Gait abnormalities	+	+	+	−	+	+
Brachydactyly	+	+	+	−	+	+
Joint pain during childhood	Unknown	Unknown	+	−	−	−
Genu varum	+	+	+	−	+	+
Restricted extension at the elbows	+	+	+	−	+	+
Scoliosis	−	Unknown	Unknown	−	Unknown	+
Lumbar lordosis	−	Unknown	Unknown	−	Unknown	-
Degenerative joint disease	+	−	−	+	−	−
Myasthenia	−	−	−	−	−	−

M, male; F, female; +, positive phenotype; −, negative phenotype.

**Figure 2:**
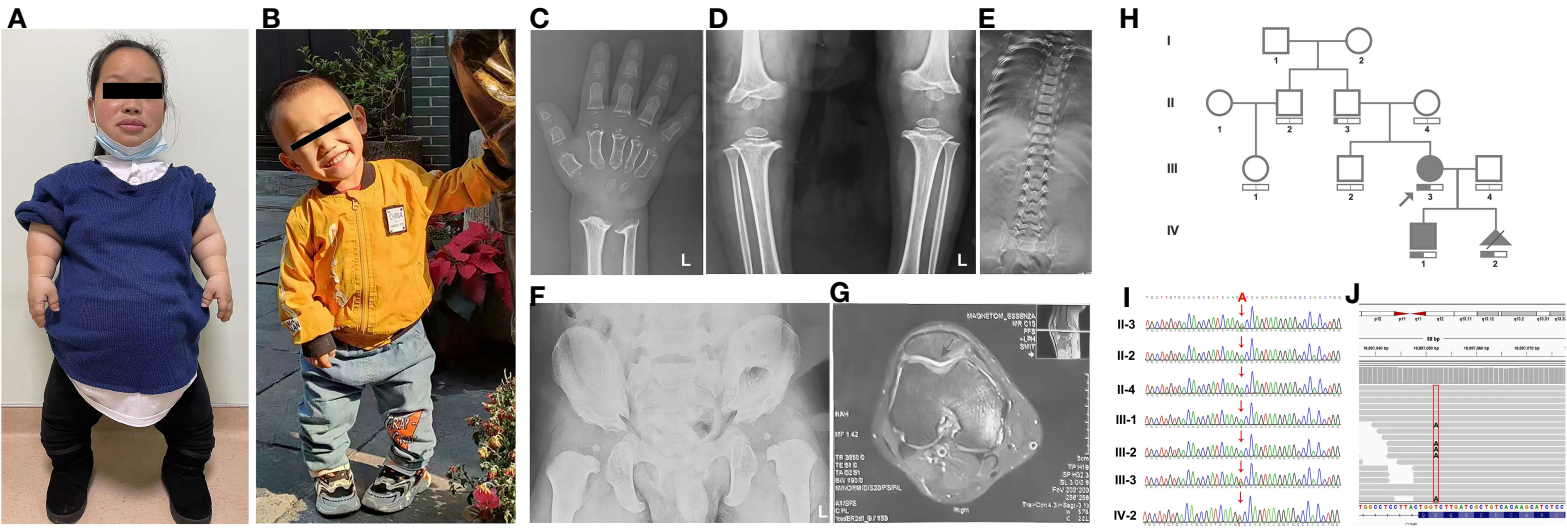
The clinical images, radiological characteristics, pedigree and co-segregation of the *COMP* mutation in family 2. **(A, B)** The clinical photographs depict short-limb dwarfism in both the proband **(A)** and her son **(B)**. **(C–G)**, Highlighting the radiological characteristics of the proband’s son. Brachydactyly of the hand, along with flared metaphyseal borders of the distal radius and ulna **(C)**. Presence of irregular and rough epiphyseal ends in long bones **(D)**. Scoliosis **(E)**. Notable findings include a shortened inferior pubic ramus, an increased pubic-ischial line width, and widening of the linea aspera of the femur **(F)**. Magnetic resonance imaging of the proband’s father indicates joint effusion **(G)**. **(H)** Pedigree of family 1. **(I)** Consequence of sanger sequencing. **(J)** For II-3, WES analysis revealed that the *c.1304A>T* variant was present in 15% of the reads.

Family 2: The proband in family 2, a 28-year-old pregnant woman with a height of 100 cm, presented for prenatal diagnosis. Her son exhibited short stature (79.5 cm) and displayed clinical features similar to those of his mother, including disproportionately short limbs, a waddling gait, genu varum, and restricted extension of the upper limbs at the elbows (**Figures 2A, B** and **Table 1**). Due to the proband’s pregnancy, she did not undergo X-ray examination. However, the X-ray results of the proband’s son revealed brachydactyly of the hands and flared metaphyseal borders of the distal radius and ulna with increased bone density (**Figure 2C**). Furthermore, irregular and rough epiphyseal ends of long bones were observed, along with widened metaphyses and smaller epiphyses in the bilateral femurs and tibias, accompanied by widened joint spaces and abnormal internal knee rotation (**Figure 2D**). Scoliosis and tongue-like projections of the vertebral bodies were also noted, although there were no signs of vertebral gap narrowing (**Figure 2E**). Moreover, there was a shortened inferior pubic ramus, an increased pubic-ischial line width, and a widening of the linea aspera of the femur (**Figure 2F**). In contrast, the proband’s father showed no significant clinical features apart from joint effusion observed during imaging (**Figure 2G**).

### 3.2 Genetic analysis

In family 1, both the proband and her fetus underwent whole-exome sequencing (WES) to identify potential pathogenic variants. Following the American College of Medical Genetics and Genomics (ACMG) guidelines, we manually evaluated all identified variants and classified them as pathogenic (P) or likely pathogenic (LP). In the proband, we discovered a novel variant in the *COMP* gene *(NM_000095.3: c.1319G>T*, p.G440V). The same variant was also present in the fetus (III-1) (**Figure 1H**), leading to the decision to terminate the pregnancy at 19 weeks. To validate the presence of the identified variant in the proband, we performed Sanger sequencing on other family members, including the proband’s sister, mother, and father (**Figure 1I**). The sequencing results confirmed that the variant co-segregated with PSACH. Specifically, all affected family members, including the proband, her sister, and her mother, carried the heterozygous *COMP* mutation, whereas the unaffected father did not.

In family 2, our investigation utilizing Trio-WES analysis unveiled a previously unreported mutation (*NM_000095.3:127 c.1304A>T*, p.D435V) within the *COMP* gene, which has not been documented in scientific literature. However, it is worth noting that this variant was submitted to ClinVar and was classified as a “Likely Pathogenic” variant by Blueprint Genetics on 13/01/2020. While the clinical assertion section specifies that this variant was identified through the Comprehensive Growth Disorders/Skeletal Dysplasias and Disorders Panel, it is crucial to emphasize the notable absence of crucial contextual information, including clinical diagnosis, clinical phenotypes, family history, and ethnicity, among other pertinent details. This deficiency in comprehensive information presents a challenge in definitively establishing a direct association between the D435V variant and PSACH. Within family 2, the D435V variant was identified in both the proband and her son (**Figure 2H**). The same mutation was also present in the fetus (IV-2), leading to the decision to terminate the pregnancy. Sanger sequencing was performed to confirm the presence of the mutation in the proband and to validate its inheritance from their father, who exhibited no significant clinical features except for joint effusion observed on imaging (**Figures 2G**, **2I**). To potentially explain the milder clinical phenotype observed in the father, WES was conducted. The results revealed that the *c.1304A>T* mutation was present in 15% of the reads (**Figure 2J**).

### 3.3 Predicting the effect of variation on protein structure and function

VarSite, Missense3D, and PyMOL were employed to evaluate the functional consequences of the G440V and D435V variants on the COMP protein. The structure of the COMP protein complex was obtained from the Protein Data Bank in Europe (PDBe), providing a basis for analysis. PyMOL was utilized to perform a comparative analysis, make necessary modifications, and visualize the molecular structures of both the wild-type and variant forms of the COMP protein. These tools facilitated a comprehensive examination of the structural changes and potential functional impacts caused by the identified variants.

**Figure 3:**
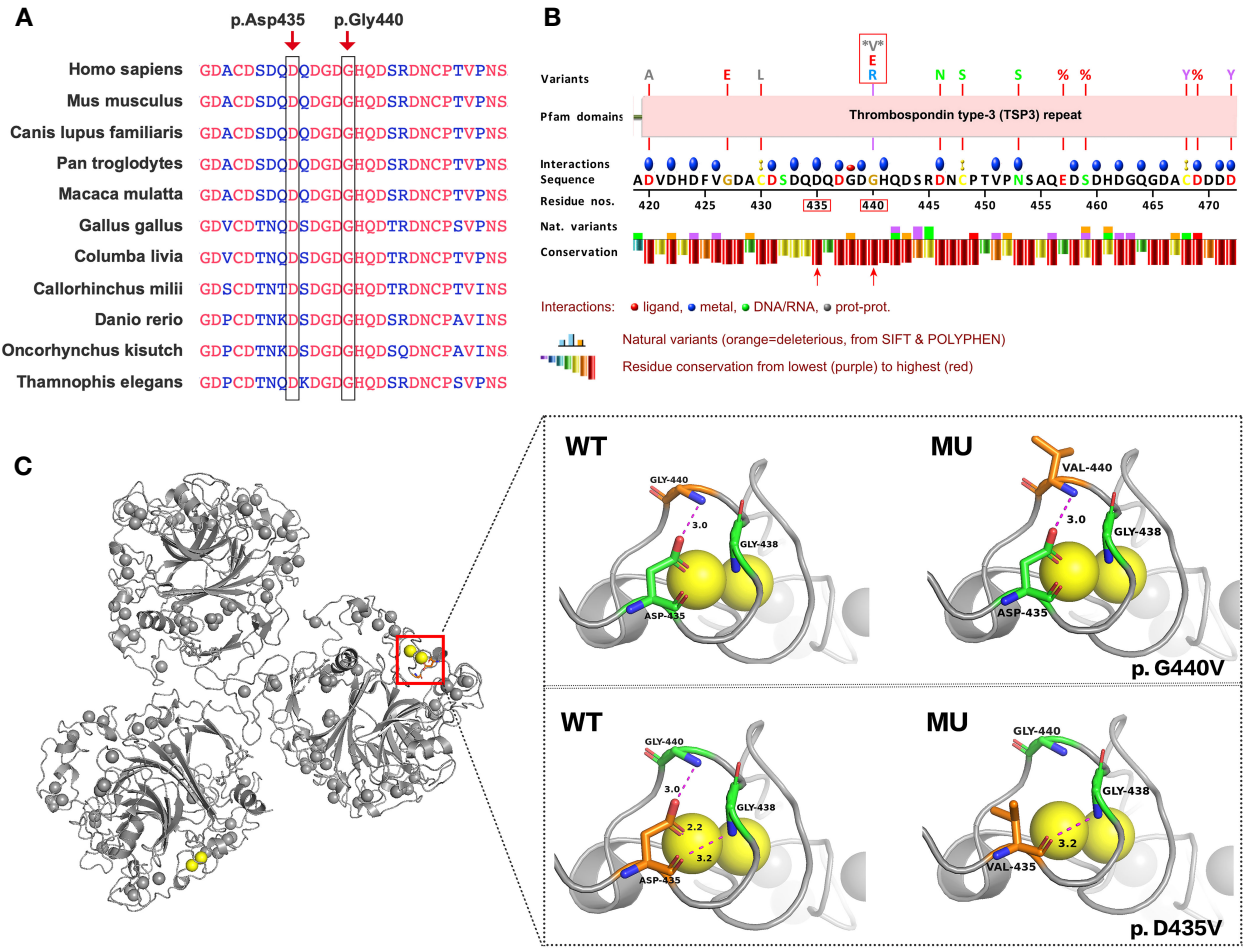
Predicting the impact of G440V and D435V variations on COMP protein’s structure and function. **(A)** G440V and D435V exhibit high evolutionary conservation across species. The black box highlights these sites across species for comparison. **(B)** The VarSite diagram illustrates a section of the COMP protein with various sequence and structural annotations. **(C)** The protein structure of COMP is depicted with and without the mutant variations. The top panel illustrates the effect of G440V, whereas the bottom panel portrays the effect of D435V. WT represents the wild-type COMP protein, whereas MU represents the mutant COMP protein.

The amino acid residues Gly440 and Asp435 exhibit high conservation across different species, as shown in **Figure 3A**. **Figure 3B** presents a schematic VarSite diagram illustrating various sequences and structural features of the COMP protein. Notably, no natural variants have been reported at position Gly440 in the gnomAD database, except for two disease-associated variants, G440G and G440E. All variants, including the newly identified G440V, have CADD scores above 25 (**Table S1**), indicating their likely deleterious nature. In contrast, Asp435 has a CADD score of 32, suggesting its potential deleterious effect (probably deleterious).

Missense3D, an online tool, was employed to assess the impact of G440V on the protein structure, as depicted in **Figure 3C** (Right Top). The Gly440 residue, situated in a bend curvature and assigned the secondary structure of “S” by the DSSP algorithm, is predicted to be detrimentally affected by the substitution of glycine with any other amino acid at this position. Furthermore, PyMOL was utilized to examine the structural consequences of D435V (**Figure 3C**, Right Bottom). The substitution of valine for aspartic acid at position 435 (D435V) disrupts the hydrogen bond between Asp435 and Gly440 residues, as well as the interaction with the calcium ion (CA 817).

## 4 Discussion

In this study, we present two families diagnosed with PSACH, which were confirmed through exome sequencing and further validated using Sanger sequencing. Through WES, we identified two novel missense variants (*c.1319G>T* and *c.1304A>T*).

The pathogenicity of *c.1319G>T* (p.G440V) is supported by various lines of evidence. Firstly, the variation occurs within the functional thrombospondin type 3 repeat domain (PM1). Secondly, the frequency of this variant is not present in the gnomAD database (PM2_supporting). Thirdly, the variant occurs at the same position as other known pathogenic missense changes, such as *c.1318G>A* (p. Gly440Arg), *c.1318G>C* (p. Gly440Arg), and *c.1319G>A* (p. Gly440Glu) (PM5). Fourthly, the variant co-segregates with PSACH in the affected families (PP1). Lastly, multiple bioinformatics tools predict deleterious effects of the mutation on COMP (PP3). Considering these factors, this variant was interpreted as a likely pathogenic variant (PM1+PM2-supporting+PM5+PP1+PP3) following the ACMG guidelines (16).

In the second case, the variant *c.1304A>T* was derived from the mosaic father of the proband. Several factors were taken into consideration to assess its pathogenicity: (1) the variation occurred within the functional thrombospondin type 3 repeat domain (PM1); (2) the absence of this variant in various reference population databases, including 1000 Genomes Project, CNGBdb, ExAC, and gnomAD, supports its rarity (PM2_supporting); (3) the variant co-segregated with the disease in affected family members (PP1); (4) multiple bioinformatics tools predicted deleterious effects on COMP (PP3); (5) the observed phenotype in this case aligns with the known disease association (PP4). Based on these considerations, this variant was interpreted as a likely pathogenic variant following the ACMG guidelines (PM1+PM2_supporting+PP1+PP3+PP4) (16).

A comprehensive study conducted by Briggs et al., which examined 300 *COMP* pathogenic variants associated with pseudohydroplasia and/or autosomal dominant multiple epiphyseal dysplasia (MED), established a robust genotype–phenotype correlation. Pathogenic missense variants predominantly associated with the pseudoachondroplasia phenotype were located in the nucleotides encoding the sixth to eighth type III calcium-binding repeats, specifically T3_6_, T3_7_, and T3_8_. In this study, two novel variants (*c.1319G>T* and *c.1304A>T*) were identified in the sixth type III calcium-binding repeat (T3_6_) of *COMP*. Moreover, our investigation unveiled the co-segregation of the variant with PSACH, as evidenced by the presence of the heterozygous *COMP* mutation in all affected family members, whereas unaffected individuals did not harbor this mutation. The clinical phenotype observed in affected individuals is consistent with the specific features of PSACH, including brachydactyly, a waddling gait, genu varum, mild thoracic deformity, and restricted extension at the elbows in the upper limbs. Additionally, in the second family, the proband’s father manifested a milder clinical phenotype, with only a joint effusion, and WES analysis revealed the presence of the *c.1304A>T* mutation in 15% of the reads. Collectively, the evidence presented in our study strongly supports the concordance between the two mutations and the well-established genotype–phenotype correlation in PSACH.

Cartilage oligomeric matrix protein (COMP) is a multimeric glycoprotein composed of five monomers which are held together by a coil–coil domain to form a pentameric structure (Posey et al.,2018). Interestingly, knockout of the *COMP* gene in mice does not result in any noticeable phenotypic changes (17). This suggests that it is the dysfunction of mutated forms of COMP, rather than a reduction in protein levels, that leads to PSACH. The concept of dominant negative was proposed by Ira Herskovitz in 1987 as an approach to studying gene function (18). In cases where a protein functions as a multimer, mutated forms of the protein may exert inhibitory effects by interacting with wild-type peptide chains, thereby resulting in the formation of non-functional multimers. Notably, Maddox et al. observed the accumulation of COMP and type IX collagen proteins on expanded rough endoplasmic reticulum vesicles in cartilage from PSACH patients, and Posey et al. simulated this process at the cellular level using a mouse model(19). The classic mutant Asp469del COMP has been well-documented to be retained in the endoplasmic reticulum (ER)(20). Similarly, the D475N mutant COMP, also located in the T3 repeat region, has been reported to exhibit ER retention (21). In contrast, mutant proteins situated in the C-terminal region, such as COMP H587R, T585M, and several other mutations within the C-terminal domain (CTD), exhibited efficient secretion into the culture media of diverse cell models. Despite their efficient secretion, these mutants continued to exert detrimental effects on cellular viability and disrupt the extracellular matrix (21, 22). Additionally, research conducted by Bell et al. has provided valuable insights into the pathogenic mechanisms. They demonstrated that mutations in matrilin-3 (Matn3 V194D) or COMP (Comp T585M and Comp DelD469) can alter the extractability of other cartilage proteins, ultimately leading to the disruption of cartilage tissue integrity. Notably, this disruption appears to be genotype-specific (23). Collectively, these findings underscore both the consistency and diversity of pathogenic mechanisms.

The Gly440 residue is originally situated within a bend curvature in the protein structure. Glycine, characterized by its small side chain, exhibits greater flexibility compared with other amino acids, allowing it to adopt a wide range of phi/psi backbone dihedral angles. This flexibility makes glycine commonly found in loops and regions where the polypeptide chain undergoes sharp turns. The absence of a bulky side chain enables glycine to occupy compact spaces and execute tight turns without encountering steric clashes. When any other amino acid replaces glycine at position 440, it can lead to an increase in energy required for conformational changes and may impact the overall stability of the protein. Consequently, such substitutions are considered damaging.

The Asp435 residue is situated within the T3 repeats, with each repeat sequence containing two DxDxDGxxDxxD motifs (11). The Asp residues within these motifs carry a negative charge, enabling them to bind to calcium ions and contribute to the structural stability of the protein. Specifically, Asp435 interacts with calcium ion CA 817 in conjunction with Asp437, Asp439, His441, and Asp446. The substitution of Asp435 with Valine (D435V) results in the elimination of the negative charge, disrupting the interaction between Asp435 and CA 817. This disruption ultimately destabilizes the protein. Maddox et al. revealed that COMP3-Mu (D446N) peptides bound less than half the amount of calcium when compared with the COMP3-WT peptides. This indicates that a single amino acid mutation at a calcium-binding site could significantly impact binding at other sites (24). Additionally, Asp435 is involved in forming hydrogen bonds with glycine (Gly) residues at positions 438 and 440. While the D435V mutation does not affect the hydrogen bond with Gly438, it does disturb the hydrogen bond with Gly440, potentially leading to structural and functional alterations in the protein. The D435V mutation not only breaks this hydrogen bond but also affects its interaction with calcium ions (CA 817). This may explain why the D435V mutation is associated with more severe clinical symptoms compared with G440V.

Taken together, it is highly probable that these two missense mutations induce endoplasmic reticulum retention, exerting a dominant negative influence on the normal function of wild-type COMP and other cartilage proteins. The inclusion of *in vitro* and animal experiments may provide valuable insights to address this question more definitively.

In summary, we have identified two novel heterozygous likely pathogenic mutations (*c.1319G>T* and *c.1304A>T*) in the *COMP* gene, which contribute to our knowledge of the genetic basis of PSACH. Our findings underscore the clinical significance of T3 repeats and calcium ions in the functioning of COMP proteins. The addition of these two novel variants to the existing spectrum of *COMP* mutations enhances our understanding of the genetic diversity underlying this disease. This information can be valuable for diagnostic testing, genetic counseling, and the development of targeted therapies for affected individuals and families.

## Data availability statement

The raw data supporting the conclusions of this article will be made available by the authors, without undue reservation.

## Ethics statement

The human samples used in this study were acquired from primarily isolated as part of our previous study for which ethical approval was obtained.

## Author contributions

LZ: Data curation, Writing – original draft, Writing – review & editing. JC: Resources, Writing – original draft. QL: Software, Writing – original draft. SY: Formal Analysis, Writing – original draft. WX: Formal Analysis, Writing – review & editing. YP: Project administration, Supervision, Writing – review & editing.
