# Supplementary material for: Case Report: Whole-exome sequencing identified two novel COMP variants causing pseudoachondroplasia
Source: Front Endocrinol (Lausanne). 2023 Nov 23;14:1267946. doi: 10.3389/fendo.2023.1267946 (PMC10702128; doi:10.3389/fendo.2023.1267946)
Supplement: Supplementary file 1 [file Table_1.docx]

Supplementary Material

## Supplementary Table 1

| **Variant** | **Chrom.** | **Coord.** | **Codon change** | **raw** | **PHRED** | **Significance** |
| --- | --- | --- | --- | --- | --- | --- |
| Gly440Val | 19 | 18786135 | gGa/gTa | 3.83 | 25.1 | likely deleterious |
| Gly440Glu | 19 | 18786135 | gGa/gAa | 3.86 | 25.2 | likely deleterious |
| Gly440Arg | 19 | 18786136 | Gga/Aga | 4.01 | 25.8 | likely deleterious |
| Gly440Arg | 19 | 18786136 | Gga/Cga | 3.98 | 25.6 | likely deleterious |
| Asp435Val | 19 | 18786242 | gAc/gTc | 4.81 | 32 | probably deleterious |
